# Silicon-nanoparticles doped biochar is more effective than biochar for mitigation of arsenic and salinity stress in Quinoa: Insight to human health risk assessment

**DOI:** 10.3389/fpls.2022.989504

**Published:** 2022-10-10

**Authors:** Hameed Alsamadany, Hesham F. Alharby, Hassan S. Al-Zahrani, Yahya M. Alzahrani, Afaf A. Almaghamsi, Ghulam Abbas, Muhammad Ansar Farooq

**Affiliations:** ^1^Department of Biological Sciences, Faculty of Science, King Abdulaziz University, Jeddah, Saudi Arabia; ^2^Department of Biology, College of Science, University of Jeddah, Jeddah, Saudi Arabia; ^3^Department of Environmental Sciences, COMSATS University Islamabad, Vehari Campus, Islamabad, Pakistan; ^4^Institute of Environmental Sciences and Engineering, School of Civil and Environmental Engineering, National University of Sciences and Technology (NUST), Islamabad, Pakistan

**Keywords:** silicon biochar, sustainability of natural resources, health risk, arsenic, Quinoa

## Abstract

The increasing contamination of soil with arsenic (As), and salinity has become a menace to food security and human health. The current study investigates the comparative efficacy of plain biochar (BC), and silicon-nanoparticles doped biochar (SBC) for ameliorating the As and salinity-induced phytotoxicity in quinoa (*Chenopodium quinoa* Willd.) and associated human health risks. Quinoa was grown on normal and saline soils (EC_e_ 12.4 dS m^−1^) contaminated with As (0, 20 mg kg^−1^) and supplemented with 1% of BC or SBC. The results demonstrated that plant growth, grain yield, chlorophyll contents, and stomatal conductance of quinoa were decreased by 62, 44, 48, and 66%, respectively under the blended stress of As and salinity as compared to control. Contrary to this, the addition of BC to As-contaminated saline soil caused a 31 and 25% increase in plant biomass and grain yield. However, these attributes were increased by 45 and 38% with the addition of SBC. The H_2_O_2_ and TBARS contents were enhanced by 5 and 10-fold, respectively under the combined stress of As and salinity. The SBC proved to be more efficient than BC in decreasing oxidative stress through overexpressing of antioxidant enzymes. The activities of superoxide dismutase, peroxidase, and catalase were enhanced by 5.4, 4.6, and 11-fold with the addition of SBC in As-contaminated saline soil. Contamination of grains by As revealed both the non-carcinogenic and carcinogenic risks to human health, however, these effects were minimized with the addition of SBC. As accumulation in grains was decreased by 65-fold and 25-fold, respectively for BC and SBC in addition to As-contaminated saline soil. The addition of SBC to saline soils contaminated with As for quinoa cultivation is an effective approach for decreasing the food chain contamination and improving food security. However, more research is warranted for the field evaluation of the effectiveness of SBC in abating As uptake in other food crops cultivated on As polluted normal and salt-affected soils.

## Introduction

Arsenic (As) is a metalloid and due to its high toxicity and widespread environmental contamination, it is categorized as a class-1 carcinogen for humans (Bhat et al., 2021; Shabbir et al., 2021). The negative effects of As are not limited to humans, but it is equally dangerous for plants. Arsenic accumulation in plants causes a decline in the uptake of essential nutrients, photosynthesis, stomatal conductance, plant growth, and grain yield (Panda et al., 2017; Shamshir et al., 2022).

Arsenic also results in oxidative stress in plants by generating reactive oxygen species (ROS) primarily, singlet oxygen (^½^*O*_2_), hydrogen peroxide (H_2_*O*_2_), superoxide (${\text{O}}_{2}^{•-}$), and hydroxyl (HO^•^) radicals (Bhat et al., 2021; Naeem et al., 2022). The most severe effects of ROS have been observed on proteins, nucleic acids, carbohydrates, and cell membranes (Shamshir et al., 2022). The toxicity induced by ROS is deterred by the activation of different antioxidant enzymes including superoxide dismutase (SOD), catalase (CAT), ascorbate peroxidase (APX), and peroxidase (POD) (Farooq et al., 2021; Shamshir et al., 2022).

Soil salinity is another threat to the environment, affecting approximately 6% of land and 20% of the irrigated area globally (Qadir et al., 2014; Abbas et al., 2022). The adverse effects of soil salinity on plants are mainly manifested as osmotic stress, ion toxicity, and nutritional imbalances. These disorders may trigger secondary metabolic changes, i.e., reduction in cell division, photosynthetic pigments, and oxidative damage leading to complete destruction of crops (Flowers and Colmer, 2015; Abbas et al., 2022). The damage caused to the plants by excessive salt concentration is dependent on exposure time, plant genotype, growth stage, and soil type (Abbas et al., 2021; Shabbir et al., 2021).

To deal with the problem of food security on saline soils, the cultivation of salt-tolerant crops such as quinoa could be one of the plausible options (Abbas et al., 2022). Quinoa is a facultative halophyte naturally equipped with the potential to grow on saline soils contaminated with heavy metals (Shabbir et al., 2021; Naheed et al., 2022), and produce seeds of exceptional nutritional quality providing countless medicinal benefits (Gaikwad et al., 2021). However, quinoa cultivation on As-contaminated saline soils does not yield desirable results because of retarded growth and grain yield apart from contamination of the food chain (Parvez et al., 2020; Shabbir et al., 2021). For addressing the increasing human population globally, it is crucial that heavy metal(loid) contamination and salt-affected soils are resolved for improving food security. The cultivation of food crops on metal-contaminated saline soils necessitates appropriate soil treatments to improve plant growth and inhibit the accumulation of toxic metals in food crops.

Biochar is a porous carbonaceous substance manufactured through the pyrolysis of biomass at different temperatures with little or no oxygen (Zama et al., 2017). The addition of biochar to saline and metal-contaminated soils has been reported to enhance plant growth and grain yield substantially (Shabbir et al., 2021; Abbas et al., 2022). Biochar is considered an excellent sorbent of various contaminants, and its tampering with various materials has been reported to improve its sorption capacity for the targeted pollutants (Zama et al., 2018). Such associations of materials with biochar result in the alteration of physicochemical properties of biochar making it a more adaptable and novel material with increased efficacy (Zama et al., 2017). Likewise, silicon (Si) plays a promising role in plant tolerance against salinity and heavy metals (Abbas et al., 2022; Zhao et al., 2022). However, until recently, no data was available regarding the effectiveness of Si-doped biochar for increasing crop growth and yield and reducing human health risks associated with quinoa consumption cultivated on As-contaminated saline soils. Hence, the current study was planned to explore the comparative efficacy of plain biochar (BC) and Si-doped biochar (SBC) for reducing As-induced phytotoxicity in quinoa grown under saline conditions and associated human health risks. It was hypothesized that SBC may be more effective than BC in reducing the buildup of As in quinoa under salinity stress. This study was envisioned to compare the efficacy of BC and SBC on the growth, physiological attributes, and grain yield of quinoa. Parallel to this, the accumulation of As by quinoa, its translocation from root to the foliage, and mitigation of human health risks caused by the consumption of quinoa grown on As-contaminated saline soils also formed a part of the key objectives of this research.

## Materials and methods

### Preparation of biochar and its characterization

Biochar was prepared using cotton stalks collected from crop fields. The stalks were air dried and pyrolyzed in a muffle furnace at 400°C (Naeem et al., 2020). The prepared BC was taken out from the furnace and stored in zipper bags. The SBC was prepared following the method described by Zulfiqar et al. (2016). The BC was doped with silicon nanoparticles by using 320 mL sodium silicate solution (SSS) in 1,600 mL distilled water. Simultaneously, a solution of acetic acid and ethanol (1:4) was prepared with 400 mL acetic acid in 1600 mL ethanol. One kg BC was thoroughly mixed in SSS to prepare a slurry-type mixture. Subsequently, this mixture was titrated against the acetic acid and ethanol solution. The resultant SBC was filtered, oven-dried, and stored for further application.

The characterization of BC and SBC for various physicochemical properties was done using standard protocols as provided in the Supplementary Table 1. Briefly, the method of Qayyum et al. (2015) was followed for the determination of the volatile organic matter both in the BC and SBC at 450°C. The surface area of biochar was determined with a Brunauer-Emmett-Teller analyzer (BET, Tristar II 3020). The ash contents were estimated as detailed by Slattery et al. (1991). The EC, pH, and CEC were determined by following Gaskin et al. (2008). The total phosphorus (P) content was estimated by the vanadate-molybdate method (Chapman and Pratt, 1961) using a UV/Vis spectrophotometer (Lambda 25, PerkinElmer, USA). The contents of potassium (K) were estimated using a flame photometer (BWB-XP5), whereas the estimation of nitrogen (N) was done by the Kjeldahl method. The contents of As and Si were determined on atomic absorption spectrophotometer (PerkinElmer Atomic Absorption Spectrophotometer pinAAcle 900F, Inc. USA). The functional groups at the surfaces of BC and SBC were explored using the Matson Polaris IR spectrophotometer. The chemical composition and surface morphology of BC and SBC were investigated using a TESCAN Vega LMU-scanning electron microscope (SEM) coupled with Energy Dispersive X-Ray Analysis (EDX). The amorphous nature of BC and SBC was revealed by X-ray diffraction (XRD) as described by Naeem et al. (2022) in unpublished data.

### Experimental setup and treatments application

Normal and saline soil (S) were sampled from two separate fields. The collected soil samples were ground, passed through a 2 mm sieve, and analyzed for physicochemical characteristics (Supplementary Table S2). The experiment was conducted in a glass house with a mean day/night temperature of 27/12°C, relative humidity of 48/74%, and day length was 8 h and 18 mins. Both the normal and saline soils were filled in pots @ 10 kg. Spiking of normal and saline soil was done using sodium arsenite (NaAsO_2_) salt for As treatments (0, 20 mg kg^−1^ soil). The BC and SBC were mixed in soil @ 1% on a dry weight basis. Each plastic pot contained 10 kg of soil. Phosphorous and nitrogen (33 and 67 mg kg^−1^ soil, respectively) were mixed in each pot using DAP and Urea as recommended by Shabbir et al. (2020). The control treatment did not receive any of these applications, i.e., salinity, biochar, and As. Two plants of quinoa genotype “Puno” were grown in each pot until maturity. All the pots were irrigated with distilled water on alternate days with the same quantity of water. All the pots were randomly arranged in the glass house to avoid any biases among various treatments. There were two plants in each replication with four replicates of each treatment. Similar studies using biochar under various heavy metals have been reported by Shabbir et al. (2021) and Naeem et al. (2022). These authors conducted pot experiments using biochar and its composites with nanoparticles under natural conditions and reported the effectiveness of biochar and its nanocomposites in reducing the toxic effects of heavy metals in plants.

### Plant growth and metal analysis

Quinoa plants were harvested at maturity and shoot and root lengths were measured. Shoot and root samples were oven-dried at 75°C till constant weight and their dry weights were recorded. The grain weight of quinoa was also noted using a digital balance. For metals analysis, oven-dried plant samples were digested in HClO_4_ and HNO_3_ by following the methods of AOAC (1990). The digestates were analyzed on the atomic absorption spectrophotometer for As determination in plant samples. The estimation of sodium (Na) and K was carried out by analyzing the samples on a flame photometer.

### Determination of plant physiological attributes

Six weeks after germination, the quinoa plants were harvested for studying their physiological attributes. The method given by Lichtenthaler (1987) was followed for the quantification of leaf pigments. Stomatal conductance was measured under bright sunlight, using a portable leaf porometer (Decagon Devices, Pullman, 142 Washington, USA). The membrane stability of quinoa leaves was assessed by measuring the electrical conductance of leaf leachate at two distinct temperatures i.e., 40°C after 30 mins and 100°C after 10 mins (Sairam et al., 2002). To quantify the relative water contents (RWC), 0.5 g leaf samples were taken and their fresh weight (FW) was recorded. These samples were placed in 100 ml DW for 4 h and their turgid weight (TW) was noted. After oven drying the samples at 70°C for 48 h, their dry weight (DW) was calculated. The RWC was calculated by using the following Equation (1) below.


(1) $$\begin{array}{l} \text{RWC}=\left(\text{FW}-\text{DW}\right)/{\left(\text{TW}-\text{DW}\right)}^{*}100 \end{array}$$

### H_2_O_2_ and lipid peroxidation

For H_2_O_2_ and lipid peroxidation essays, fresh leaf samples (0.5 g) were homogenized in trichloroacetic acid and centrifuged at 12,000 × g for 20 mins, and H_2_O_2_ contents were determined as detailed by Islam et al. (2008). While lipid peroxidation analysis was conducted by weighing 0.5 g leaf sample from each replication and homogenized at 4°C in hydro-alcoholic solution (80/20: v/v). After homogenizing the mixture, Thiobarbituric acid (TBA) and butyl hydroxytoluene (BHT) were added to the samples and incubated at 95°C. After incubation, the samples were centrifuged at 12,000 × g for 10 mins. The contents of Thiobarbituric acid reactive substances (TBARS) were estimated by following the methods of Hodges et al. (1999).

### Determination of antioxidant enzymes

The enzymatic activities in plant leaves were determined by taking 250 mg fresh leaf samples and grinding in 0.1 M phosphate buffer maintained at pH 7.0. The mixture of plant samples was centrifuged at 15,000 × g for 30 mins. The activities of catalase (CAT), superoxide dismutase (SOD), and peroxidase (POD) were estimated by the methods devised by Dhindsa et al. (1981), Aebi (1984), and Hemeda and Klein (1990), respectively.

### Translocation factor, bioconcentration factor, and tolerance index

The ratio of As concentration in the shoot to root was used to calculate translocation factor (TF). The As concentration in plant tissues was divided by As in soil to calculate the bioconcentration factor (BCF). The dry weight of metal-stressed plants was divided by the dry weight of non-stressed plants to get tolerance index (TI) (Shabbir et al., 2020).

### Estimation of human health risks

Following the guidelines given by U.S. EPA (2011), the health risks to humans ingested with quinoa grown on saline soils contaminated with heavy metals were determined as per Equation (2) below.


(2) $$\begin{array}{l} \text{EDI }=\text{ AT }\times \text{ Cg }\times \text{ ED}/\text{BW }\times \text{ IR }\times \text{ EF} \end{array}$$

Where EDI stands for estimated daily intake of As (mg kg^−1^ day^−1^), AT is the average life expectancy. Cg is representing the concentration of As in grains, while ED is the exposure duration (70 years globally). EF denotes the exposure frequency, BW is the average body weight (70 kg) and IR is the ingestion rate of quinoa for children (16 g) and adults (25 g) day^−1^ (Li et al., 2018), respectively.

Incremental Lifetime Cancer Risk (ILTCR) was estimated according to Equation (3) below (Shabbir et al., 2021).


(3) $$\begin{array}{l} \text{ILTCR}=\text{CSF }\times \text{ EDI} \end{array}$$

Where CSF is the cancer slope factor, which is 1.5 mg kg^−1^ day^−1^ for As (U.S. EPA, 2012).

The hazard quotient (HQ) or non-cancerous hazard related to the consumption of quinoa grains was estimated as described by Rehman et al. (2016).


(4) $$\begin{array}{l} \text{HQ}=\text{EDI}/\text{RfD} \end{array}$$

Where RfD reflects the oral reference dose of As and its value is 0.0003 mg kg^−1^ day^−1^.

### Statistical analyses of the data

The analysis of the data was done by two-way analysis of variance (ANOVA), at a significance level of 5%, using Statistix 8.1 software. The least significant difference (LSD) test was used for the comparison of the treatments along with the standard error of means (Steel et al., 1997).

## Results

### Characterization of biochar

The characterization of biochar (Supplementary Table 1) indicated that SBC had greater volatile matter (41.47 vs. 27.25%) and ash contents (57.33 vs. 42.52%) than BC. However, the organic carbon contents were less in SBC as compared to BC. The decrease in organic carbon fraction in SBC may be attributed to an increase in Si contents which were infused on BC (Ahmad et al., 2017). The CEC of the SBC (17.23 c mol_c_ kg^−1^) was greater than that of BC (9.46 cmol_c_ kg^−1^). The SBC contained more K and less N and P than BC, whereas total carbon was higher in BC (54.4%) as compared to SBC (51.6%). The Si contents of BC and SBC were 3.0 and 17.5 mg g^−1^ respectively. The SBC exhibited a porous network due to Si doping and it caused a substantial increase in the BET surface area of BC (5.37–28.43 m^2^ g^−1^).

The EDX analysis revealed the absence of Si in BC, but considerably higher levels (6.9%) in SBC proved that SBC was successfully impregnated with silicon nanoparticles (Supplementary Figure S1). The surface of SBC showed more heterogeneity and porosity than BC, which could potentially assist As immobilization by SBC as compared to BC. The morphology of biochar was explored by scanning electron microscopy (SEM) (Supplementary Figure S1). The SEM analysis indicated that BC and SBC exhibited porous, active, and well-distributed surfaces. Due to increased porosity, the adsorption potential of the particles was increased. The difference in pore space between BC and SBC is responsible for the difference in the adsorption potential of biochar. The SBC displayed the existence of SiNPs, which developed more micro-openings and improved the Si-adsorption potential of SBC as compared to BC.

The XRD spectra of BC and SBC revealed the occurrence of several mineral phases (Supplementary Figure S2A). The SBC contained more crystalline Si phases, with higher intensity peaks at 26.65°, 57.86°, and 72.60° which can be attributed to SiO_2_ (quartz) (Xu et al., 2017). Additionally, calcite could also be responsible for projecting a peak at 39.57° in SBC and 40.64° in BC (Zama et al., 2018). In comparison to the BC, these peaks decreed the existence of Si in SBC.

The presence of organic functional groups on the surfaces of BC and SBC was explored through the FTIR technique (Supplementary Figure S2B). Transmission from 4,000–3,000 cm^−1^ represents OH bonds due to organic or inorganic components, whereas the transmission at 2,916 cm^−1^ both in BC and SBC indicated C–H symmetric stretching vibration in organic carbon (Kumar and Rajkumar, 2014). The results indicated that double and triple bond stretching (C=O, –C=C–, and –C=N) occurred in the 2,000–2,400 cm^−1^ transmission. The peak at 1,250–1,565 cm^−1^ both in BC and SBC indicated the presence of C-C and C–C bond stretching due to aromatic rings. While –C–O is responsible for the SBC peak maxima at 1,312–1,368 cm^−1^. The peaks obtained at 782–1,282 cm^−1^ for C O, C-O, Si–O–Si, and ${\text{PO}}_{4}^{3-}$ on SBC were corresponding to enhanced phosphate and silicate buildup. Sharp peaks for SBC represented the Si–O–Si group at 1,082 and 782 cm^−1^ (Xiao et al., 2014). Similarly, the peaks at 1,070 cm^−1^ indicated C-Si bonds for SBC (Zama et al., 2018). In SBC, spectrum, transmission in the range of 657 and 782 cm^−1^ were corresponding to Si–O, and Si–O–Si symmetric peaks, respectively (Li et al., 2018). Notably, these peaks were either missing or appeared very weak for BC.

### Biomass and grain yield of Quinoa

Results revealed that plant growth and grain yield of quinoa were significantly less on saline and As-contaminated soils. The highest reduction in plant biomass and grain yield was reported on As-contaminated saline soil as is evident from the data in Table 1. The biomass of plant roots, foliage, and grain yield declined by 20, 25, and 15%, respectively on saline soil compared with plants grown on normal soil (control). Contamination of soil with As caused a respective decrease of 37, 30, and 25%, in shoot and root biomass and grain yield of quinoa plants compared to those grown on normal soil. However, on As-contaminated saline soil, the decreases in the shoot (62%), root (56%), and grain yield (44%) were considerably higher compared with plants grown and maintained on normal soil. As expected, the addition of BC and SBC to saline and As-contaminated soil significantly increased plant growth. For instance, shoot and root dry weights and grain yield were noted as 31, 37, and 25% higher when As-contaminated saline soil was augmented with BC. On the other hand, the addition of SBC to As-contaminated saline soil resulted in a 45, 51, and 38% enhancement of dry weights of shoot, root, and grains compared to As-contaminated saline soil without BC.

**Table 1:** Effect of salinity, arsenic, and their combination on growth and grain yield of quinoa supplemented with no biochar (NBC), biochar (BC), and silicon-nanoparticles doped biochar (SBC).

**Biochar types**	**Salinity and As treatments**	**Shoot dry weight (g plant^−1^)**	**Root dry weight (g plant^−1^)**	**Grain weight (g plant^−1^)**	**Shoot length (cm)**	**Root length (cm)**
NBC	C	8 ± 0.3 ab	5 ± 0.2 b	8 ± 0.3 c	29 ± 1.0 b	25.5 ± 1.0 b
	S	6 ± 0.2 c	4 ± 0.2 d	6.8 ± 0.2 e	21.2 ± 1.1 c	20 ± 0.8 c
	As	5 ± 0.4 de	3.5 ± 0.1 e	6 ± 0.2 f	13 ± 0.9 de	16 ± 0.9 d
	S-As	3 ± 0.3 f	2.2 ± 0.2 f	4.5 ± 0.3 g	9 ± 0.8 f	12 ± 0.5 e
BC	S	7 ± 0.2 bc	5 ± 0.3 b	8 ± 0.2 c	26 ± 1.2 bc	24 ± 1.2 b
	As	6.5 ± 0.3 c	4.6 ± 0.4 c	7.2 ± 0.3 d	23 ± 1.3 c	21 ± 0.9 c
	S-As	4.4 ± 0.25 e	3.5 ± 0.2 e	6 ± 0.4 f	12.5 ± 0.5 e	17 ± 0.5 d
SBC	S	8.5 ± 0.4 a	6 ± 0.3 a	9.5 ± 0.3 a	33.1 ± 1.5 a	27 ± 0.7 a
	As	7.8 ± 0.2 b	5.7 ± 0.2 ab	8.5 ± 0.2 b	28.8 ± 0.8 b	25 ± 0.6 b
	S-As	5.5 ± 0.2 d	4.5 ± 0.1 c	7.2 ± 0.3 d	17.2 ± 1.0 d	21 ± 0.5 c

The values (average ± SE of four replicates) within each column followed by different letters represent the significant difference at a significance level of 5%.

Salinity and As impaired the shoot growth of quinoa plants thereby declining the shoot length up to 26, 55, and 68% under salinity, As, and their combined application respectively. However, biochar had an ameliorating effect, where the addition of BC caused a 28 and 29% increase in the growth of shoots and roots with respect to As-contaminated saline soil without biochar. Quinoa plants growing on SBC treated As-contaminated saline soil exhibited a 48 and 43% increase in shoot and root growth in contrast to the soil without SBC.

### Physiological attributes

Stomatal conductance, chlorophyll, and relative water contents of quinoa leaves were significantly diminished due to salinity, As, and their combined application (Table 2). Stomatal conductance of experimental plants was 22, 44, and 66% lesser under salinity, As, and their combined treatment compared to control. Similarly, compared to control, relative water contents were decreased by 16, 24, and 41% under salinity, As, and their combined application, so also leaf pigments revealed a reduction of 17, 23, and 48% under saline conditions, As and their combined applications. The BC-treated As-contaminated saline soil showed a respective increase of 25, 17, and 19% in stomatal conductance, chlorophyll, and relative water contents, compared to those plants on untreated soil. On the other hand, supplementation of SBC in As-contaminated saline soil caused increases of 48, 29, and 31% in stomatal conductance, chlorophyll contents, and relative water contents of quinoa in comparison to the soil without SBC.

**Table 2:** Effect of salinity, arsenic, and their combination on physiological attributes of quinoa supplemented with no biochar (NBC), biochar (BC), and silicon-nanoparticles doped biochar (SBC).

**Biochar types**	**Salinity and As treatments**	**Stomatal conductance (mmol m^−2^ s^−1^)**	**Total chlorophyll (μg g^−1^ FW)**	**Relative water contents (%)**
NBC	C	360 ± 10 b	480 ± 12 a	85 ± 3.5 a
	S	280 ± 8 d	400 ± 9 d	71 ± 2 c
	As	200 ± 5 f	370 ± 8 e	65 ± 2.5 d
	S-As	120 ± 11 h	250 ± 10 g	50 ± 3 f
BC	S	330 ± 5 c	450 ± 11 b	80 ± 2 b
	As	240 ± 8 e	415 ± 15 c	76 ± 3 bc
	S-As	160 ± 10 g	300 ± 12 f	62 ± 2 e
SBC	S	370 ± 6 a	485 ± 10 a	84 ± 2.2 a
	As	300 ± 7 d	460 ± 9 b	79 ± 1.8 b
	S-As	230 ± 8 ef	350 ± 10 e	72 ± 1.9 c

The values (average ± SE of four replicates) within each column followed by different letters represent the significant difference at a significance level of 5%.

### Na and K contents in plant tissues

**Figure 1:**
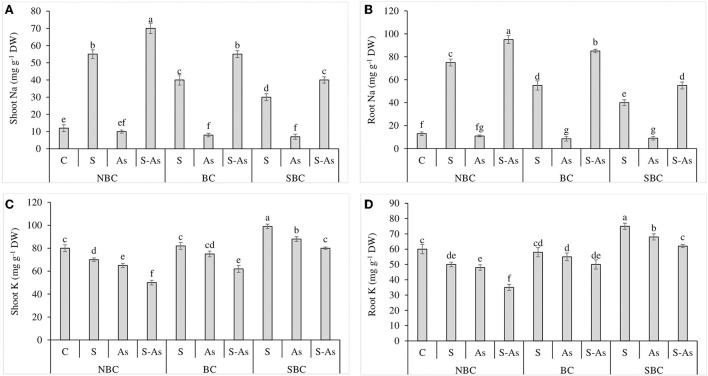
Effect of salinity, arsenic, and their combination on shoot Na **(A)**, root Na **(B)**, shoot K **(C)**, and root K concentration **(D)** of quinoa supplemented with no biochar (NBC), biochar (BC), and silicon-nanoparticles doped biochar (SBC). The values (average ± SE of four replicates) followed by different letters represent the significant difference at a significance level of 5%.

The concentrations of Na in shoot and root tissues of quinoa were considerably enhanced by 4.5 and 5.7-fold under salt stress conditions compared to control. However, under the combination of As and salinity, shoot and root accumulated 5.8 and 7.3-fold higher Na compared with normal soil (Figures 1A,B). The addition of SBC was more effective than BC in limiting the accumulation of Na in the under and above-ground parts of quinoa. The addition of BC in S-As treatment caused 4.58 and 6.5-fold enhancement in Na contents in the shoot and root of quinoa. However, the accumulation of Na in the shoot and root was 3.3 and 4.2-fold higher under the application of SBC, in comparison to an unamended S-As soil.

The buildup of K in shoot and root decreased significantly under salinity, As, and their combination in comparison to control (Figures 1C,D). Potassium (K) concentration in the plant shoot was reduced by 13, 19, and 38% under saline stress, As, and their combined treatment as compared to control, while the concentration of K in the root decreased by 17, 20, and 42% under salinity, As, and their combined treatment with respect to normal soil. The augmentation of As-contaminated saline soil with BC caused a significant increase of 19 and 30% in K accumulation in the shoot and root respectively while these improvements were noted as 38 and 44%, respectively when growth mediums were supplemented with SBC in contrast to unamended soil.

### Arsenic accumulation and translocation

**Figure 2:**
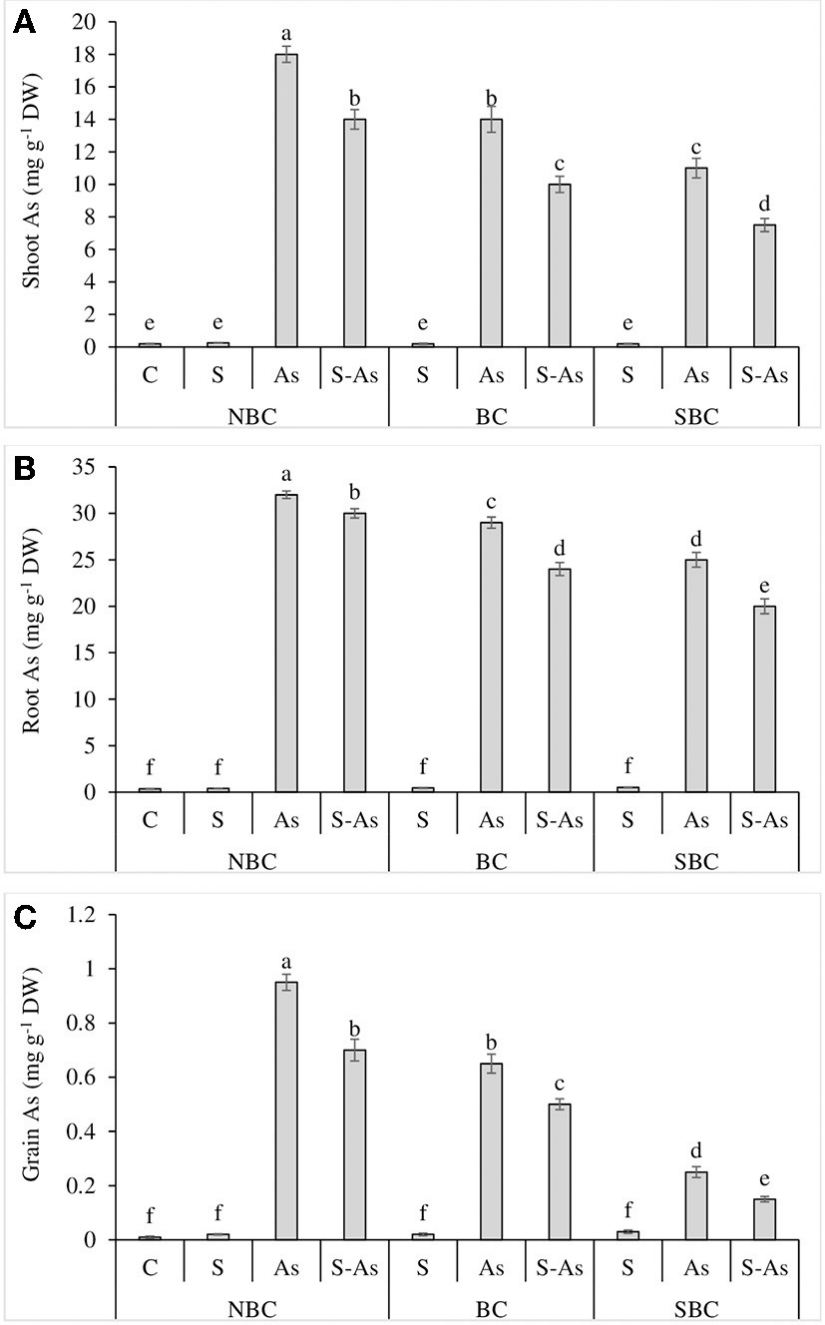
Effect of salinity, arsenic, and their combination on shoot As **(A)**, root As **(B)**, and grain As **(C)** concentration of quinoa supplemented with no biochar (NBC), biochar (BC), and silicon-nanoparticles doped biochar (SBC). The values (average ± SE of four replicates) followed by different letters represent the significant difference at a significance level of 5%.

**Figure 3:**
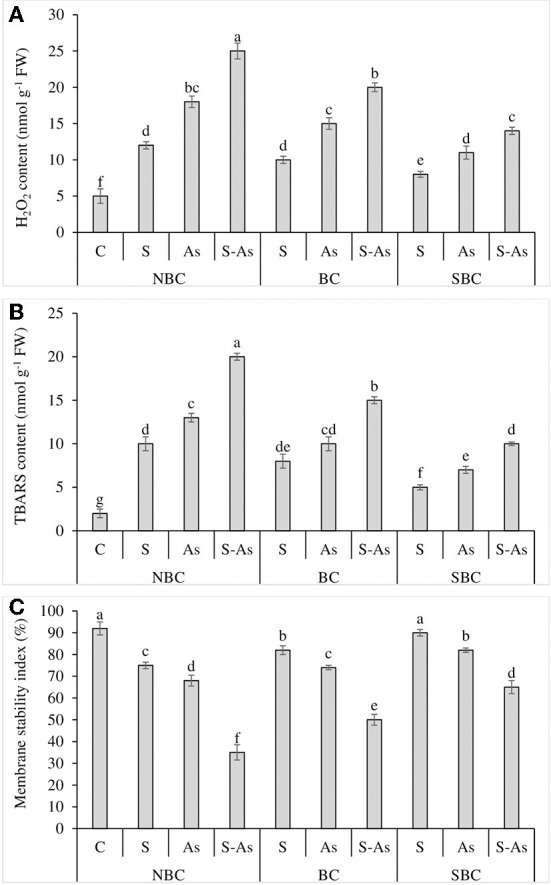
Effect of salinity, arsenic, and their combination on H_2_O_2_ contents **(A)**, TBARS contents **(B)**, and membrane stability index **(C)** of quinoa supplemented with no biochar (NBC), biochar (BC), and silicon-nanoparticles doped biochar (SBC). The values (average ± SE of four replicates) followed by different letters represent the significant difference at a significance level of 5%.

Whilst comparing the As accumulation in different plant parts, it was observed that the highest amount of this metalloid was accumulated in roots and the lowest in grains. The highest accumulation of As was reported in the shoot, root, and grains of quinoa plants grown in As-contaminated soil without salinity (Figures 2A–C). The concentrations of As in root, shoot, and grains of quinoa were enhanced by 91, 90, and 95-fold under As-contaminated soil compared to control (Figures 1B, 3A). The application of SBC to the growth medium reduced the buildup of As in root, shoot, and grains of quinoa more significantly in comparison to the BC application. The addition of BC in As treatment caused only 82, 70, and 65-fold enhancement in As concentration of root, shoot, and grains of quinoa. However, As content in the root, shoot, and grain was 71, 55, and 25-fold higher under the application of SBC, in contrast to unamended As-contaminated soil. The value of BCF was greater than one both under sole application of As as well as under combined application of As and salinity (Table 3). Surprisingly, the alteration of As-contaminated soil with SBC reduced the BCF <1. A similar response was observed in the case of TF, where it was reported to be <1 for As alone treatment and its combination with salinity, while the addition of SBC to As-contaminated soil further lessened the TF value. The values of TI were 75, 63, and 38%, under the respective treatments of salinity, As, and a combination of these. The addition of BC and SBC to saline soil contaminated with As resulted in the respective increase of 55 and 69% in TI in comparison to the soil without biochar amendments.

**Table 3:** Effect of salinity, arsenic, and their combination on bioconcentration factor (BCF), translocation factor (TF), and tolerance index (TI) of quinoa supplemented with no biochar (NBC), biochar (BC), and silicon-nanoparticles doped biochar (SBC).

**Biochar types**	**Salinity and As treatments**	**BCF**	**TF**	**TI (%)**
NBC	C	–	–	–
	S	–	–	75 ± 3.0 d
	As	1.25 ± 0.02 a	0.56 ± 0.02 a	62.5 ± 2.0 e
	S-As	1.1± 0.04 b	0.46 ± 0.01 b	37.5 ± 2.0 g
BC	S	–	–	87.5 ± 3.5 c
	As	1.075 ± 0.04 b	0.48 ± 0.02 b	81.3 ± 3.0 cd
	S-As	0.85 ± 0.03 c	0.42 ± 0.01 c	55 ± 1.5 f
SBC	S	–	–	106 ± 2.1 a
	As	0.9 ± 0.06 c	0.44 ± 0.02 c	97.5 ± 3.2 b
	S-As	0.6875 ± 0.04 d	0.38 ± 0.01 d	68.8 ± 3.0 d

The values (average ± SE of four replicates) within each column followed by different letters represent the significant difference at a significance level of 5%.

### Manifestation of oxidative stress

Quinoa plants exposed to salinity, As, and their combined application grieved from oxidative stress due to an increase in H_2_O_2_ and TBARS contents as compared to plants grown on normal soil (Figures 3A,B). The contents of H_2_O_2_ were enhanced many folds under salinity, As, and/or their combination as compared to the control treatment. Likewise, the TBARS were enhanced by 5, 6.5, and 10-fold under salinity stress, As, and their combined treatment with respect to control treatment. The supplementation of S-As soil with BC caused up to a 4 and 7.5-fold increase in H_2_O_2_ and TBARS contents in quinoa. However, H_2_O_2_ and TBARS contents were only 2.8 and 5-fold higher under the application of SBC in comparison to soil receiving no SBC. The oxidative stress caused by salinity, As and their combination led to a corresponding decline in MSI, where it was observed as 18, 26, and 62% less under salinity, As, and their combined application as compared to control soil (Figure 3C). The MSI was enhanced by 30% and 46% with the respective addition of BC and SBC in As-contaminated saline soil in comparison to the soil medium having no BC and SBC additions.

### Enzymatic activities

**Figure 4:**
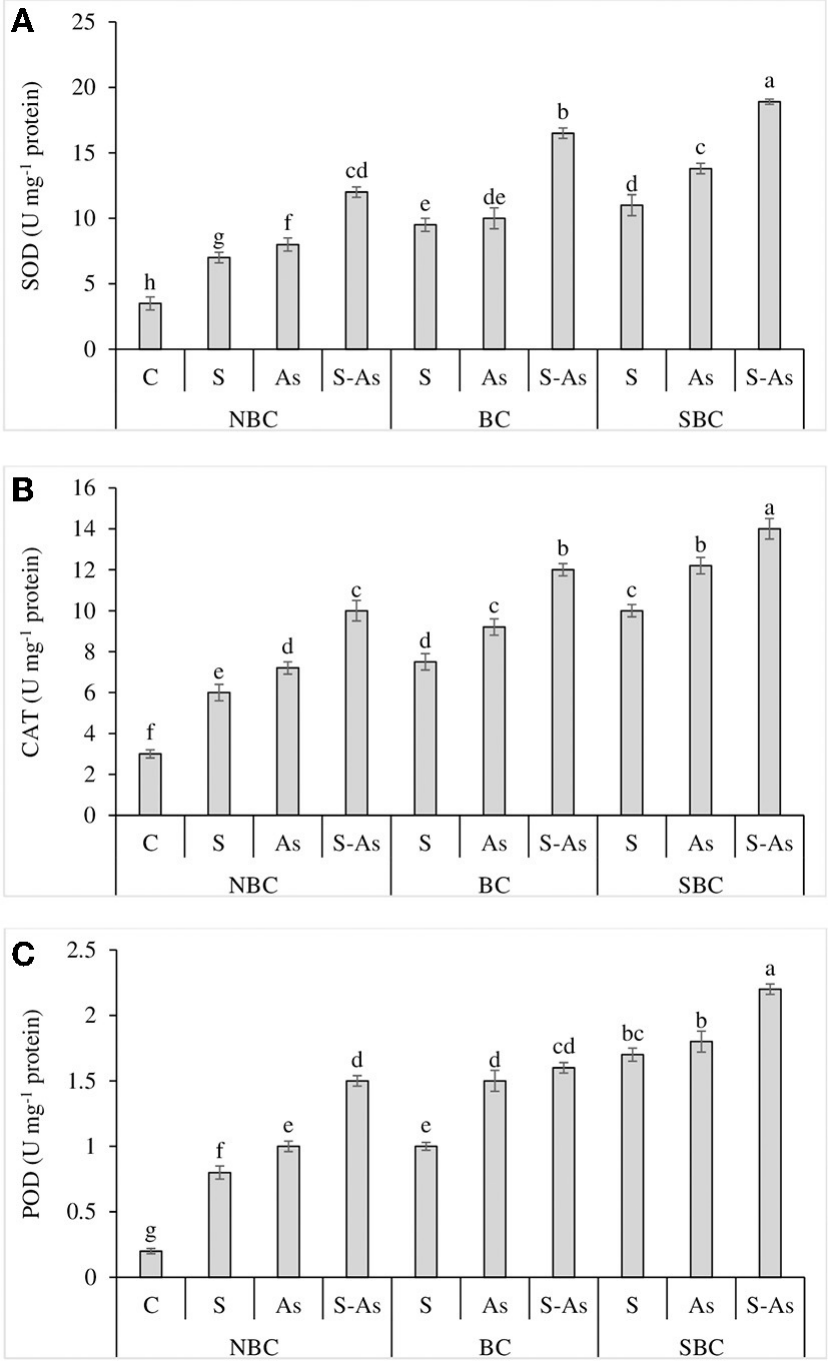
Effect of salinity, arsenic, and their combination on the activities of SOD **(A)**, CAT **(B)**, and POD **(C)** of quinoa supplemented with no biochar (NBC), biochar (BC), and silicon-nanoparticles doped biochar (SBC). The values (average ± SE of four replicates) followed by different letters represent the significant difference at a significance level of 5%.

To ameliorate the oxidative stress, the activities of antioxidant enzymes; SOD, CAT, and POD were significantly increased when quinoa plants received the combined treatment of As and salinity than individual applications of As and salinity (Figures 4A–C). There was a 2, 2.3, and 3.4-fold increase in the activity of SOD under salinity, As and their combined treatment respectively as compared to control. The activity of CAT was overexpressed by 2, 2.4, and 3.3-fold. A similar observation was recorded for POD, where the activity of this enzyme was enhanced by 4, 5, and 7.5-fold under saline stress, As, and their combination as compared to control. The application of SBC was more effective than BC in further increasing the activities of these enzymes. With the addition of BC in S-As treatment, the activities of SOD, POD, and CAT were improved by 4.7, 4, and 8-fold, respectively. However, activities of SOD, POD, and CAT were boosted by 5.4, 4.6, and 11-fold with the addition of SBC in S-As treatment in contrast to the soil without biochar.

### Health risk assessment

The HQ and ILTCR were higher than their respective threshold limits both for adults and children when quinoa was grown on As-contaminated soil (Table 4). The addition of BC decreased the value of HQ to the safe value of <1. However, the values of ILTCR were still higher than the safe limit (<0.0001). The addition of SBC caused a significant reduction in As accumulation in the grain which lessened the HQ and ILTCR values much below their threshold levels.

**Table 4:** Effect of salinity, arsenic, and their combination on health risk attributes of quinoa supplemented with no biochar (NBC), biochar (BC), and silicon-nanoparticles doped biochar (SBC).

**Biochar types**	**Salinity and As treatments**	**Adult**			**Child**		
		**EDI**	**CR**	**HQ**	**EDI**	**CR**	**HQ**
NBC	C	0.00000	0.000005	0.01190	0.0000	0.00000	0.0010
	S	0.00001	0.000011	0.02381	0.0000	0.00001	0.0227
	As	0.00034	0.000509	1.13095	0.0003	0.00049	1.0803
	S-As	0.00025	0.000375	0.83333	0.0002	0.00036	0.7960
BC	S	0.00001	0.000011	0.02381	0.0000	0.00001	0.0227
	As	0.00023	0.000348	0.77381	0.0002	0.00033	0.7392
	S-As	0.00018	0.000268	0.59524	0.0002	0.00026	0.5686
SBC	S	0.00001	0.000016	0.03571	0.0000	0.00002	0.0341
	As	0.00009	0.000134	0.29762	0.0001	0.00013	0.2843
	S-As	0.00005	0.000080	0.17857	0.0000	0.00001	0.0145

## Discussion

The results of the current investigation demonstrated that As contamination in soil severely impacted the plant growth and grain yield of quinoa without biochar application (Table 1). In line with the current results, several other studies also noted that plant biomass was drastically impaired due to As stress (Ahmad et al., 2020; Shamshir et al., 2022). Growth and biomass reduction of plants due to their exposure to As may be attributed to the limited nutrient uptake (Abbas et al., 2018), inhibition of gaseous exchange (Siddiqui et al., 2020), oxidative stress (Bhat et al., 2021; Shamshir et al., 2022), and ion toxicity (Allevato et al., 2019; Shabbir et al., 2021) caused by As.

The combined treatment of As and salinity was even more detrimental to plant growth, perhaps the excessive buildup of As and Na disturbed water balance, limited photosynthesis, and damaged the cell membrane (Panda et al., 2017; Shabbir et al., 2021). The supplementation of biochar, especially SBC, ameliorated the As-induced phytotoxicity in quinoa both under normal and saline soil environments. Previous studies have also reported the efficacy of biochar for reducing the phytotoxicity induced by As (Shabbir et al., 2021; Abbas et al., 2022; Naeem et al., 2022 and the references therein). Improved plant growth with biochar application can be attributed to the increased cationic exchange capacity (CEC) and pH of the soil, improved nutritional status and water holding capacity of the soil, and adsorption of the metal ions (Mansoor et al., 2020; Naeem et al., 2022). Moreover, due to higher CEC, biochar has a great adsorption capacity, porosity, and surface area, which helps in lessening the metal (As) and salt stress either by releasing beneficial ions such as Ca^2+^, Mg^2+^, and K for example, or by adsorbing toxic ions (Shabbir et al., 2021; Naeem et al., 2022). The doped biochar (SBC) overwhelmingly improved the plant growth and grain yield of quinoa more than undoped biochar (BC). These observations corroborate with the findings of Zama et al. (2018), who demonstrated that biochar amended with Si was more efficient than plain biochar, where the former influenced the growth of spinach grown under As stress as compared with plain or unamended biochar. Silicon-triggered growth augmentation under salt stress is related to the regulation of antioxidant enzymes, nutrient uptake, modulation of soil pH, and changes in metal speciation (Soundararajan et al., 2014). Silicon limits the metal stress by changing the soil pH or metal speciation and formation of inorganic crystals in biochar and increasing the contents of Si within plant tissues (Debona et al., 2017; Zama et al., 2018; Alharby et al., 2022).

The combined treatment of As and salinity increased the concentration of Na in quinoa (Figure 1). Na ions are sequestered in the vacuole rather than being eliminated by roots (Shabala et al., 2013). Because the ionic radius and hydration energy of Na and K are the same, hence, under salinity stress, Na enters the plant cells *via* K channels (Marschner, 1995). Salinity and As together decreased the uptake of K in quinoa tissues (Shabbir et al., 2021; Abbas et al., 2022). The outcomes of this study demonstrated the positive role of biochar, as it hindered the accumulation of Na and improved the uptake of K by quinoa plants. Thus, the use of biochar is an effective approach for reducing the harmful effects of As and salinity on plants by reducing toxic ions uptake and increasing the uptake of essential plant nutrients (Mansoor et al., 2020; Shabbir et al., 2021). Silicon doped biochar was even more effective in limiting the accumulation of toxic Na ions and it increased the accumulation of essential K ions by quinoa. It has been well established that Si-enriched biochar restricts the toxic ions (Na), increases the uptake of essential ions (K) due to the increased nutrient status of the soil, and improves root penetration. Moreover, numerous carboxylic and phenolic groups on the surface of Si biochar increase the cation exchange and water-holding capacity of the soil (Rafi et al., 2022; Zhao et al., 2022). From the current and previous research findings, it can be concluded that the addition of SBC to the salt and As-affected soils for quinoa cultivation could yield promising results as compared to the plain biochar because the former was more effective for limiting the Na absorption by plants and enhancing plant growth. However, the obtained results are under pot culture conditions, hence, they are not very conclusive. The addition of SBC to saline and As-contaminated soil under real field conditions is needed for the validation of the current results. Additionally, the results may vary with the type and extent of salt-affected soils and feedstock used for the preparation of biochar.

Physiological traits including stomatal conductance, chlorophyll, and RWC were considerably decreased when plants were exposed to the combined treatment of salinity and As. The addition of SBC improved these physiological attributes (Table 2). These results stand at par with previous research where biochar amplified these attributes in various plant species exposed to salt or metal contamination (Shabbir et al., 2021; Naeem et al., 2022; Rafi et al., 2022). The water use efficiency of plants was improved under biochar amendment leading to an increase in relative water contents of leaves (Naeem et al., 2020). In addition to this, BC and SBC treatments decrease the bulk density and enhance water holding capacity along with soil nutrient status which positively influenced plant growth and development (Tanure et al., 2019; Naeem et al., 2022).

A higher amount of As was accumulated by roots followed by shoots and grains in experiments plants (Figure 3). This observation stands in agreement with our previous experiments (Alam et al., 2019; Shabbir et al., 2021), where a significantly higher concentration of As was retained in roots as compared to the above-ground foliage of quinoa. Moreover, the As uptake in plants was reduced under saline conditions. These findings are supported by Parvez et al. (2020) who found that salinity suppressed the accumulation of As in quinoa plants. Arsenic uptake by plants is dependent on redox potential, the presence of other salts, and soil pH (Shabbir et al., 2021). According to Parvez et al. (2020), As accumulation is reduced due to the complex formation of As and Cl under salt stress. On the other hand, Bhat et al. (2021) reported that the increased accumulation of As by roots of plants might be due to increased phytochelatin development which leads to the formation of phytochelatin-As complexes. Arsenic absorption and accumulation in different parts of quinoa plants were significantly reduced when biochar was added to the growth medium. Our findings are consistent with earlier research, which revealed that biochar may immobilize As in soil and reduce its absorption by plants (Shabbir et al., 2021; Naeem et al., 2022). The reduction in As accumulation in quinoa tissues might be due to higher immobilization of As with organic matter leading to lower amounts of As available for plants (Marmiroli, 2020). Alternatively, As forms compounds with organic carbon and becomes inaccessible for plants (Beesley et al., 2013). Biochar amendments can limit the As absorption through direct or indirect interactions (Kumar and Bhattacharya, 2022). The direct interaction may include the ion exchange, electrostatic attraction, complexation, and precipitation (Hina et al., 2019), while mineral dissolution, soil pH, soil organic carbon, and CEC may be indirectly involved in limiting the As absorption (Mansoor et al., 2020). The SBC was more effective in decreasing the accumulation of As by quinoa perhaps due to the additional benefits of Si on the above-reported soil attributes (Zama et al., 2018; Rafi et al., 2022; Zhao et al., 2022). Reduced accumulation of As after Si treatments might be due to the competition between H_3_AsO_3_ and H_4_SiO_4_ for the same transport channels (Ma et al., 2008; Lee et al., 2014). The addition of SBC reduced As accumulation in quinoa due to the preferential uptake of Si (Zama et al., 2018). Furthermore, the higher immobilization of As by SBC as compared to BC is attributed to the higher surface area, porosity, heterogeneity, and more functional groups on SBC in contrast to BC (Zama et al., 2018; Naeem et al., 2022). So, it can be concluded that doping biochar with silicon is a promising approach for reducing As accumulation, phytotoxicity, and its subsequent propagation across the food chain. The SBC, in particular, limited the uptake of As in the quinoa plant leading to better growth and grain yield.

The BCF for As was >1 in the absence of SBC amendments. However, the addition of SBC decreased the value to <1. Similarly, the TF for As was <1, and biochar addition further reduced it. If the values of BCF and TF are greater than one, it is considered a strong indicator of the metal accumulator behavior of plants (Marrugo-Negrete et al., 2015). However, if both BCF and TF are <1, as is the case in our study, it reflects the phytostabilization potential of plants for As-contaminated soils (Shabbir et al., 2021). The addition of SBC caused a further reduction in BCF and TF, highlighting that the addition of Si biochar should be practiced on As-contaminated soils to limit the mobility of this carcinogen to above-ground foliage of plants.

According to our findings, the combined treatment of salinity and As caused significantly higher oxidative damage than their sole treatments (Figure 2). Increased levels of TBARS and H_2_O_2_ in quinoa plants, as well as the lower membrane integrity, were the signs of oxidative stress (Parvez et al., 2020; Shabbir et al., 2021). In line with these findings, it was also observed that As and salinity resulted in oxidative stress and membrane damage in quinoa (Shabbir et al., 2021). However, the addition of Si-augmented biochar (SBC) relieved the plants from As and salinity stress. The contents of H_2_O_2_ and TBARS were less, which resulted in better stability of cell membranes in the presence of SBC. The H_2_O_2_ is converted into the hydroxyl anion, which is even more phytotoxic (Siddiqui et al., 2020; Naeem et al., 2022). The detoxification of ROS is carried out by various antioxidant enzymes within plant organelles (Shabbir et al., 2021; Abbas et al., 2022; Naeem et al., 2022).

Under As or salt stress, antioxidant enzymes are overproduced to reduce the levels of ROS (Bhat et al., 2021; Shabbir et al., 2021). It was found that under As and salinity stress, SOD activity was enhanced. The superoxide radicals ${\text{O}}_{2}^{•-}$) are converted into hydrogen peroxide (H_2_O_2_) in the presence of SOD (Abbas et al., 2022). Arsenic and salt stress increased the activities of CAT and POD in quinoa. These enzymes are involved in the conversion of H_2_O_2_ into molecular oxygen and water. Surprisingly, the addition of biochar under salt and As stress resulted in a greater increase in antioxidant activities. The SBC further increased the activities of antioxidants than undoped biochar. Few other studies have also reported the positive role of biochar in increasing the antioxidant activities in plants growing on soils contaminated with As or salinity (Shabbir et al., 2021; Abbas et al., 2022). The results demonstrated that biochar amendments improved the plant response to oxidative stress by advancing the antioxidant activities of enzymes and increasing the plant yield under the multiple stresses of salinity and As.

Previous research revealed that consumption of As-contaminated food may cause several disorders including lung cancer, skin rashes, and kidney damage (Edirisinghe and Jinadasa, 2019). Various risk assessment methods are used for predicting human health risks from exposure to different heavy metals. In the present study, the HQ, ILTCR, and EDI were estimated for adults and children through the consumption of As-contaminated quinoa grains. It was discovered that after consuming As-contaminated grains of quinoa grown without biochar, the values of HQ and ILTCR were higher than their respective threshold limits. Although the addition of BC to the growth medium lowered the values of HQ to the safe level (<1) yet, the cancer risk value (ILTCR) was higher than the harmless limit (<0.0001). However, the addition of SBC caused a significant reduction in the As buildup in grains and lessened the HQ and ILTCR values below their threshold limits. This is the very first study demonstrating that the health risks due to the intake of As-contaminated grains of quinoa can be minimized with the addition of SBC. The results established that the addition of SBC was more effective than simple biochar for increasing plant growth, grain yield, and reducing As-related hazards to human health. However, the mitigation of health risks associated with the consumption of contaminated grains of quinoa needs further validation using SBC on As-contaminated saline soils under field conditions.

## Conclusion

To the best of our knowledge, this is the first study comparing BC and SBC for reducing As and salinity-induced phytotoxicity in quinoa and associated human health risks. It was found that plant growth and physiological attributes of quinoa growing on As-contaminated soils were severely hampered which led to a drastic reduction in grain yield of this pseudo cereal. Nonetheless, augmenting the As-contaminated saline soil with biochar improved the physiological attributes and grain yield of quinoa. The SBC was more effective than undoped biochar for ameliorating the salt and As-induced phytotoxicity in quinoa grown under the individual or combined stresses of salinity and As. The SBC lessened the accumulation of Na and As in the experimental plants. Furthermore, the transfer of As from root to shoot was reduced in the presence of SBC resulting in the As phytostabilization. Si-doped biochar (SBC) minimized the non-carcinogenic and carcinogenic human health risks posed by As-contaminated quinoa grains. Augmenting the As-contaminated saline soils with 1% SBC is a very effective technique for quinoa cultivation, with improved plant growth and lesser human health risks. Nevertheless, future studies are needed for the evaluation of the effectiveness of SBC in abating As uptake in other food crops cultivated on As-polluted, normal, and salt-affected soils under field conditions.

## Data availability statement

The original contributions presented in the study are included in the article/Supplementary material, further inquiries can be directed to the corresponding author/s.

## Author contributions

Conceptualization: HAls, HAlh, GA, and MF. Methodology: HAls, HAlh, HA-Z, YA, and AA. Software and project administration: HAls and HAlh. Validation: HA-Z, GA, and MF. Formal analysis: HAls, HAlh, HA-Z, and AA. Investigation: HAlh, YA, GA, and MF. Resources: HAlh, HA-Z, and AA. Data curation: HAls, HAlh, and YA. Writing—original draft preparation: GA and MF. Writing—review and editing: HAls, HAlh, and AA. Visualization: HAlh, GA, and MF. All authors have read and agreed to the published version of the manuscript.

## Funding

This project was funded by the Deanship of Scientific Research (DSR) at King Abdulaziz University (KAU), Jeddah, Saudi Arabia, under Grant No. RG-16-130-43.

## Conflict of interest

The authors declare that the research was conducted in the absence of any commercial or financial relationships that could be construed as a potential conflict of interest.

## Publisher's note

All claims expressed in this article are solely those of the authors and do not necessarily represent those of their affiliated organizations, or those of the publisher, the editors and the reviewers. Any product that may be evaluated in this article, or claim that may be made by its manufacturer, is not guaranteed or endorsed by the publisher.
