# Supplementary material for: Silicon-nanoparticles doped biochar is more effective than biochar for mitigation of arsenic and salinity stress in Quinoa: Insight to human health risk assessment
Source: Front Plant Sci. 2022 Oct 10;13:989504. doi: 10.3389/fpls.2022.989504 (PMC9592068; doi:10.3389/fpls.2022.989504)
Supplement: Supplementary file 1 [file Data_Sheet_1.docx]

Supplementary Material

**Supplementary Table 1**: Physicochemical characteristics of BC and SBC.

| **Parameters** | **Biochar (BC)** | **Silicon nanoparticles doped biochar (SBC)** |
| --- | --- | --- |
| pH | 8.40 (± 0.09) | 8.05 (±0.11) |
| ECe (dS m^−1^) | 1.80 (± 0.18) | 2.10 (± 0.11) |
| CEC (cmolc kg^-1^) | 9.46 (± 0.85) | 17.23 (± 1.2) |
| Ash content (%) | 42.52 (± 1.98) | 57.33 (± 1.76) |
| Volatile matter | 27.35 (± 0.85) | 39.47 (± 1.25) |
| Organic carbon (%) | 37.87 (± 2.41) | 17.40 (± 1.30) |
| Phosphorous (g kg^−1^) | 3.25 (± 0.19) | 2.97 (± 0.14) |
| Nitrogen (g kg^−1^) | 16.30 (± 1.05) | 14.00 (± 1.55) |
| Potassium (g kg^−1^) | 21.5 (± 2.05) | 27.5 (± 2.33) |
| As (mg kg^−1^) | 1.47 (±0.09) | 0.67 (±0.07) |
| Si (mg g^−1^) | 3.00 (± 1.05) | 17.50 (± 1.50) |
| Average size (nm) | 11.53 (± 1.16) | 28.33 (± 1.75) |
| Surface area (m^2^ g^−1^) | 5.37 (± 1.25) | 28.43 (± 2.05) |
| Pore volume (cm^3^ g^−1^) | 0.011 (±0.001) | 0.023 (±0.002) |
|  |  |  |

The values are mean of three replicates ± standard deviation.

**Table S2.** Physiochemical properties of experimental soils.

| Soil characteristics | Units | Non-saline soil | Saline soil |
| --- | --- | --- | --- |
| Texture |  | Sandy clay loam | Sandy clay loam |
| pH_s_ |  | 8.43 ± 0.74 | 8.51 ± 0.71 |
| EC_e_ | d S m^-1^ | 1.44 ± 0.02 | 12.4 ± 0.05 |
| SAR | (mmol L^-1^)^1/2^ | 3.25 ± 0.06 | 11.4 ± 0.05 |
| Organic matter | % | 0.41 ± 0.04 | 0.40 ± 0.04 |
| Total N | mg kg^−1^ | 7.35 ± 0.11 | 6.12 ± 0.30 |
| Available P | mg kg^−1^ | 5.20 ± 0.24 | 4.85 ± 0.22 |
| Extractable K | mg kg^-1^ | 103 ± 3.20 | 81.0 ± 5.16 |
| Total As | mg kg^-1^ | 0.21 ± 0.03 | 0.21 ± 0.04 |

^The values are mean of three replicates ± standard deviation^


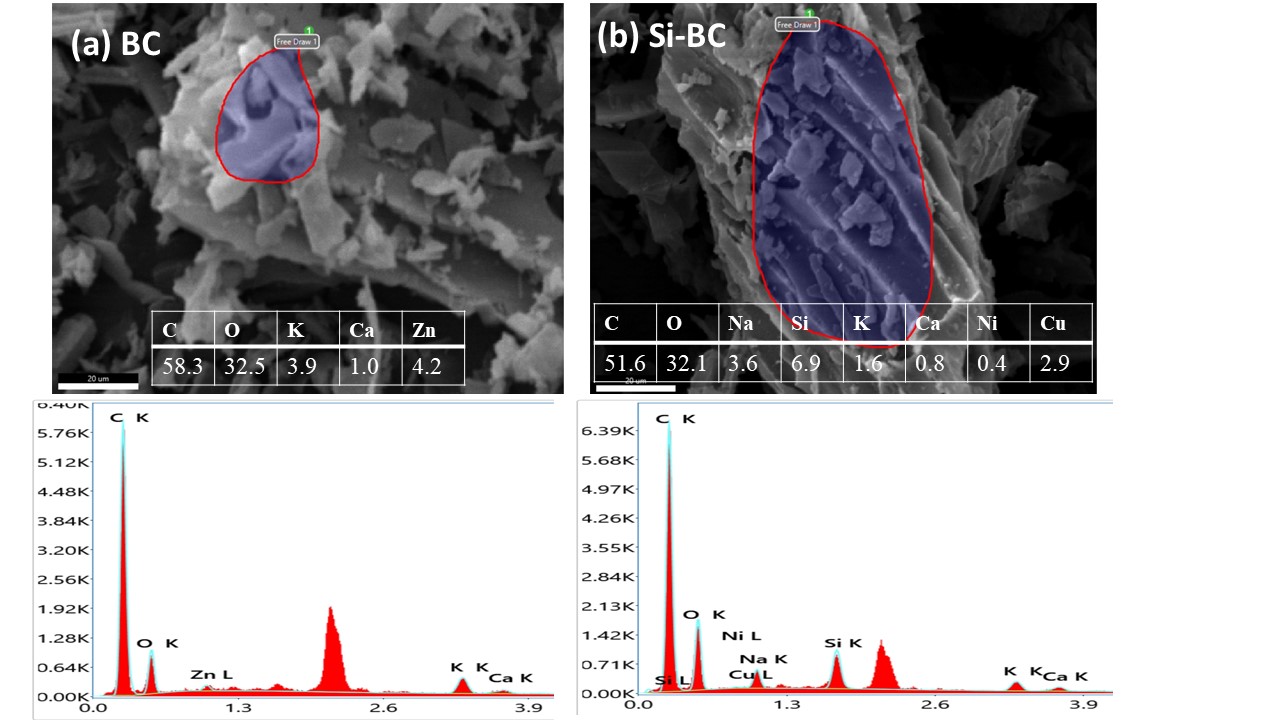


**FIGURE S1** SEM-EDX analysis of biochars representing the changes in elemental contents and morphology. The composition of elements in biochar (BC) (A), the composition of elements in silicon nanoparcticles doped biochar (SBC) (B).


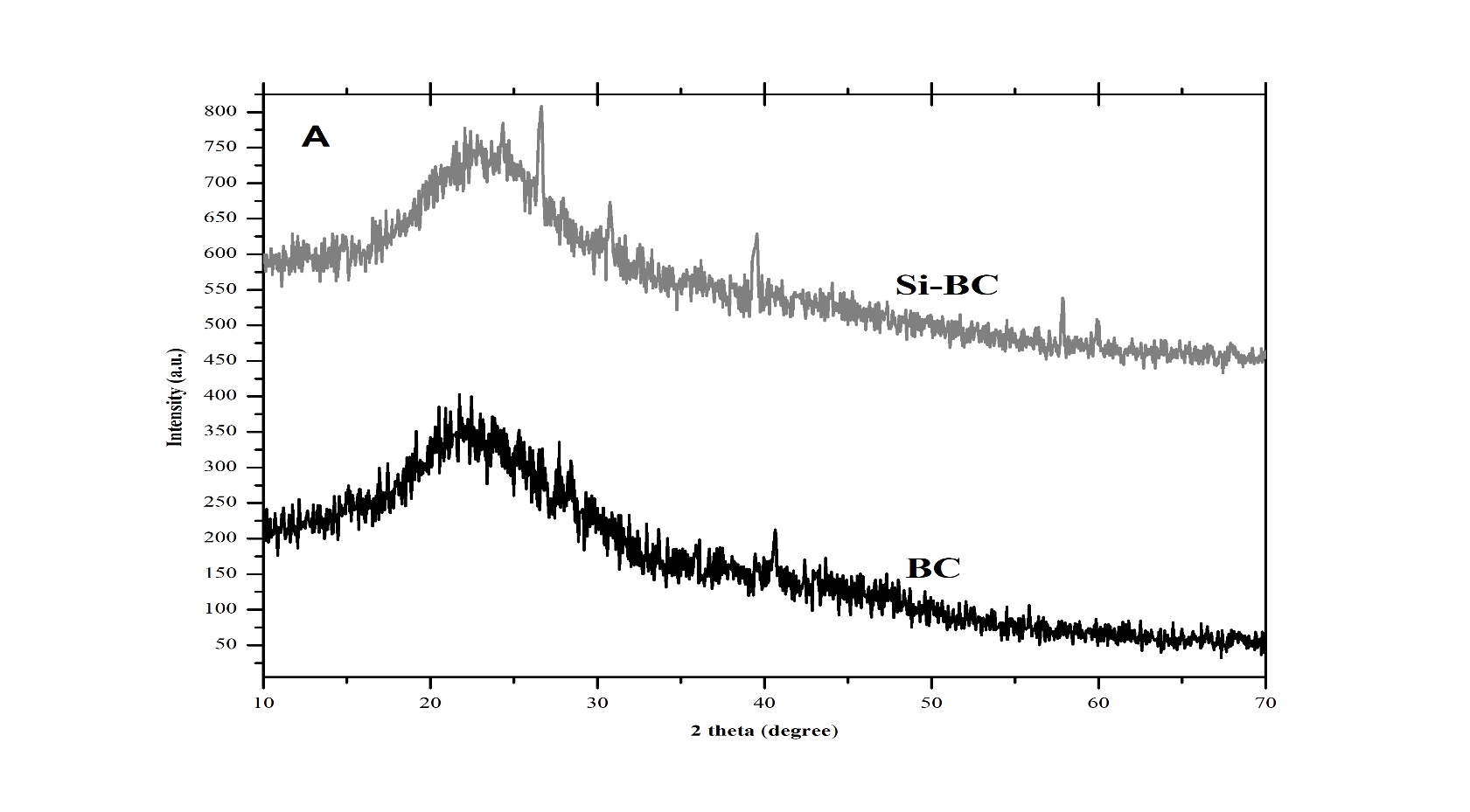


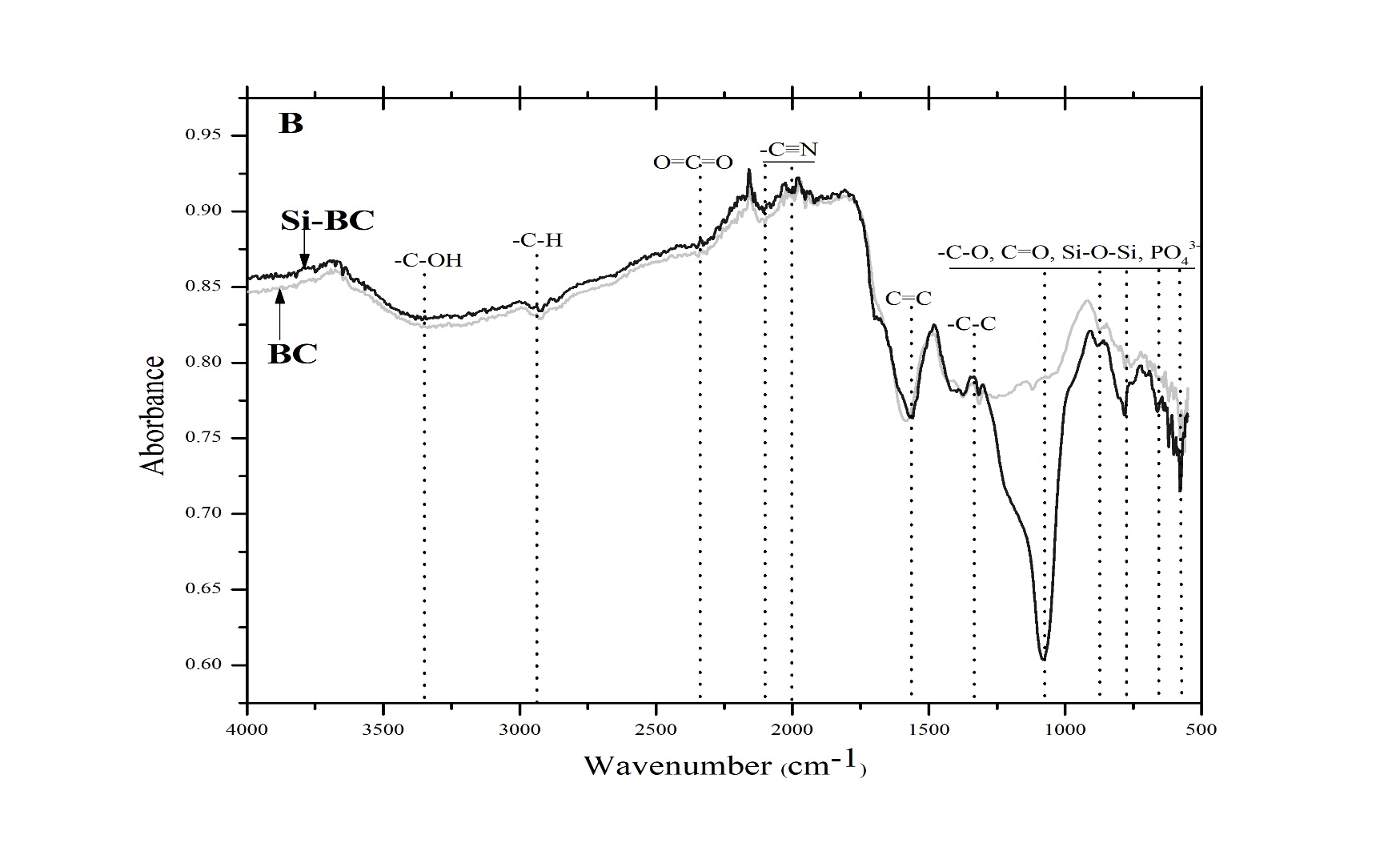


**FIGURE S2** XRD analysis of the various crystalline faces on biochar (BC) and silicon nanoparticles doped biochar (SBC) (A). FTIR analysis of bond stretching in biochar (BC) and silicon nanoparticles doped biochar (SBC) (B).
